# Effects of Oat Bran Addition on the Growth Performance and Intestinal Health of Nile Tilapia (*Oreochromis niloticus*) Exposed to Copper Ions

**DOI:** 10.1155/2023/5329546

**Published:** 2023-06-20

**Authors:** Chunze Guo, Yingchao Zhang, Xuekai Wang, Kuikui Ni, Qiang Hao, Zhen Zhang, Zhigang Zhou, Fuyu Yang

**Affiliations:** ^1^College of Grassland Science and Technology, China Agricultural University, Beijing 100193, China; ^2^College of Life Science, North China University of Science and Technology, Tangshan 063210, China; ^3^Key Laboratory for Feed Biotechnology of the Ministry of Agriculture and Rural Affairs, Feed Research Institute, Chinese Academy of Agricultural Sciences, Beijing 100081, China

## Abstract

This study investigated the effects of the oat bran addition on the growth performance and intestinal health of Nile tilapia (*Oreochromis niloticus*) exposed to copper ions. Four groups of diets containing 0%, 5%, 10%, and 20% oat bran were fed to Nile tilapia for four weeks. The results showed that oat bran had a dose-dependent effect on the growth performance of Nile tilapia. The addition of oat bran can increase the relative abundance of *Delftia*, which is capable of degrading heavy metals in the intestinal tract and alleviating the intestinal damage caused by copper ion stress. Compared to the control group, the 5% oat bran group had an increased intestinal antioxidant capacity. The relative gene expression of proinflammatory factors (NF-*κ*B, IL-1*β*) was significantly downregulated in the 5% oat bran group (*P* < 0.05), and the relative gene expression of anti-inflammatory factors (TGF-*β*), HIF-1*α*, occludin, and claudin was significantly upregulated (*P* < 0.05). In conclusion, we suggest that 5% oat bran should be added to the diet to improve the growth performance of Nile tilapia and alleviate the negative effects of copper ion stress on intestinal health.

## 1. Introduction

Pollution of the aquatic environment can affect fish health [1]. With the rapid development of industry and agriculture, a large number of heavy metal pollutants are released into the water, which causes serious water pollution. Fish are exposed to contaminated water, where heavy metals interact with biological systems, causing biochemical disturbances. Among all kinds of heavy metals, copper is one of the most common environmental pollutants in the world. Copper is an important trace element involved in many biological processes. However, copper ions at high concentrations in water can be toxic to aquatic life [2]. Studies have shown that the accumulation of copper ions in fish can induce antioxidant damage and stress responses in tissues and organs and affect their growth, development, and reproduction [3].

Diet is one of the main factors affecting the host's health [4]. When aquatic organisms are under adverse environmental stress, adopting dietary strategies can help mitigate the damage to the organisms. Oat bran, a byproduct of oat seeds, has been widely reported in food processing and human health due to its rich dietary fibre content [5–9], and it also plays a positive role in livestock and aquaculture. There have been numerous studies on the positive effects of oat bran on livestock and poultry production [10–13]. In aquaculture, Arnesen found that the amount of oat bran had a significant effect on the weight gain in rainbow trout, with a maximum occurring when oat bran was 170 g/kg in the daily diet [14]. Peiro et al. pointed out that supplementation with oat bran as a substrate had positive effects on the productive response of shrimp during their pregrowth [15].

Nile tilapia (*Oreochromis niloticus*) is recommended as a bioindicator species for environmental monitoring [16]. It has relatively strong adaptability to the environment [17]. Therefore, when environmental conditions cause stress to tilapia, the impact on its health is relatively small compared to other species. Meanwhile, Nile tilapia (*Oreochromis niloticus*) are omnivorous and can be fed plant-based diets [18]. Considering the above factors, we conducted this experimental study to elucidate that dietary supplementation with oat bran can be used as an optimal nutrition strategy to promote fish growth. At the same time, this study confirms that oat bran can be used as an immune activator to promote the intestinal health of fish, providing a novel solution to improve the healthy development of aquaculture.

## 2. Materials and Method

### 2.1. Experimental Diets

The composition of the basal diet is shown in Table 1. Four isonitrogenous and isolipidic diets were formulated to include oat bran at 0, 5, 10, and 20%. All the ingredients were mixed directly and then pelleted through a laboratory pellet machine with a 1 mm diameter; the pellets were air-dried at room temperature and then stored in plastic bags at −20°C.

### 2.2. Feeding Procedure

All experiments and animal care procedures were approved by the Feed Research Institute of the Chinese Academy of Agricultural Sciences chaired by the China Council for Animal Care (Assurance No. 2019-AF-FRI-CAAS-001). The experimental Nile tilapia fingerlings were purchased from a farm (Hainan, China) and transported to the International Agricultural High-tech Industry Park (Hebei, China), Chinese Academy of Agricultural Sciences. The purchased Nile tilapia fingerlings were temporarily raised in circulating water for two weeks before the experiment. The fish were weighed at the start of the experiment. Nile tilapia fingerlings (average initial weight 1.10 ± 0.00 g) were randomly allocated into twelve 90 L tanks. Each treatment included 3 replicate tanks. Fish were fed to apparent satiation three times daily at 8:00, 13:00, and 17:00, respectively. During the trial, the water temperature was 26°C; the pH was 7.0-7.2; the dissolved oxygen was >6.0 g/L; the total ammonia was <0.01 mg/L. No copper ions were added to the water during the first 3 weeks. Four concentrations (0 mg/L, 0.1 mg/L, 0.25 mg/L, and 0.5 mg/L) of copper ions were used for a stress test on Nile tilapia before the experiment. When the copper ion concentration was ≥0.25 mg/L, the swimming and feeding behaviors of Nile tilapia were inhibited, which indicated that the growth and health of Nile tilapia would be adversely affected when the concentration of copper ions was ≥0.25 mg/L. So in the fourth week, the copper sulfate solution was added to adjust the concentration of copper ions in the water to 0.25 mg/L. Food intake and fish deaths were counted every day.

### 2.3. Growth Measurements and Sampling

The fish were weighed and measured after fasting for 24 hours in the fourth week.

The intestine samples of Nile tilapia were collected after 24 h fasting. The intestinal content samples of Nile tilapia were collected after 6 h fasting. The samples for histological analysis were immediately placed in 4% paraformaldehyde, and the remaining samples were immediately stored in liquid nitrogen for subsequent analysis.

### 2.4. Histological Analysis

Three fish from each treatment group with 3 replicates were taken for gut histological analysis. The samples were embedded in paraffin, sectioned, stained with hematoxylin-eosin (H&E), and observed by microscope (Leica DMIL-LED, Germany).

### 2.5. Biochemical Parameters and Enzyme Analysis

Total antioxidant capacity (T-AOC), superoxide dismutase (SOD), catalase (CAT), and malonaldehyde (MDA) levels in the intestine were measured using diagnostic reagent kits following the manufacturer's (Beyotime Biotechnology, Shanghai, China) instructions. The activity of lysozyme (LYS) and the content of C3 and C4 in the intestine were tested using assay kits (Meimian, China).

### 2.6. Quantitative Real-Time PCR Analysis

Twelve fish from each treatment group were randomly sampled to obtain 6 replicates for gut cytokine gene expression analysis. The TRIzol method was used to extract total RNA from the intestinal samples. 1 *μ*g RNA was taken from each sample and transformed into cDNA. SYBR Green Premix Ex Taq™ II (TaKaRa) was used for RT-qPCR using cDNA as a template. The detailed methods were described in the literature [19]. Expressions of occludin, claudin, hypoxia-inducible factor 1 subunit alpha (HIF-1*α*), nuclear factor kappa-B (NF-*κ*B),interleukin 1 beta (IL-1*β*), interleukin 6 (IL-6), interleukin 10 (IL-10), and transforming growth factor beta (TGF-*β*) were determined using qPCR. The primers are listed in Table 2[20]. *β*-Actin was used as the reference gene, and data were analyzed according to the 2^-*ΔΔ*CT^ method [21].

### 2.7. Evaluation of Gut Microbiota

Gut microbiota of Nile tilapia in the experiment was analyzed using 16S rRNA gene sequencing. 4 h after the last feeding, the intestinal content samples of Nile tilapia fingerlings were collected from 12 fish in each treatment group to get 6 replicates. All obtained sequence datasets have been uploaded to the NCBI Sequence Read Archive (SRA) with the accession number PRJNA937597. DNA extraction, sequencing, and data analysis were conducted according to the method previously described by Zhang et al. [22].

### 2.8. Statistical Analysis and Calculation

The weight gain ratio (WGR, %), feed coefficient ratio (FCR), specific growth rate (SGR, %), and survival rate (SR, %) were calculated as follows:

(1) $$\begin{array}{c} \text{WGR}\left(\%\right)=100\times \frac{\text{final body weight}\left(\text{g}\right)–\text{initial body weight}\left(\text{g}\right)}{\text{initial body weight }\left(\text{g}\right)},\\ \text{FCR}=\frac{\text{total dry feed intake}\left(\text{g}\right)}{\text{final body weight}\left(\text{g}\right)–\text{initial body weight}\left(\text{g}\right)},\\ \text{SGR}\left(\%/\text{day}\right)=100\times \frac{\ln\text{ final body weight}\left(\text{g}\right)–\ln\text{ initial body weight}\left(\text{g}\right)}{\text{days}},\\ \text{SR }\left(\%\right)=100\times \frac{\text{final fish number}}{\text{initial fish number}}. \end{array}$$

Data were expressed as mean ± SEM (standard error of mean). SPSS 22.0 (SPSS Inc., IL, USA) and GraphPad Prism 5.0 (GraphPad Software Inc. CA, USA) software were used for data statistics and analysis. The effects of each diet were compared using a one-way analysis of variance followed by Duncan's post hoc test. The difference was considered as significant when *P* < 0.05.

## 3. Results

### 3.1. Growth Performance and Feed Utilization

After four weeks of feeding with the experimental diets, the final body weight, weight gain ratio, feed coefficient ratio, specific growth rate, and survival rate of Nile tilapia were tested, and the results are presented in Table 3. Compared with the control group, fish fed diets supplemented with 5% and 20% oat bran exhibited no significant difference in any growth performance or feed utilization indicators (*P* > 0.05, Table 3). Moreover, FBW, WGR, and SGR were significantly increased in the 10% OB group compared with those in the control group (*P* < 0.05, Table 3).

### 3.2. Intestinal Morphology

**Figure 1:**
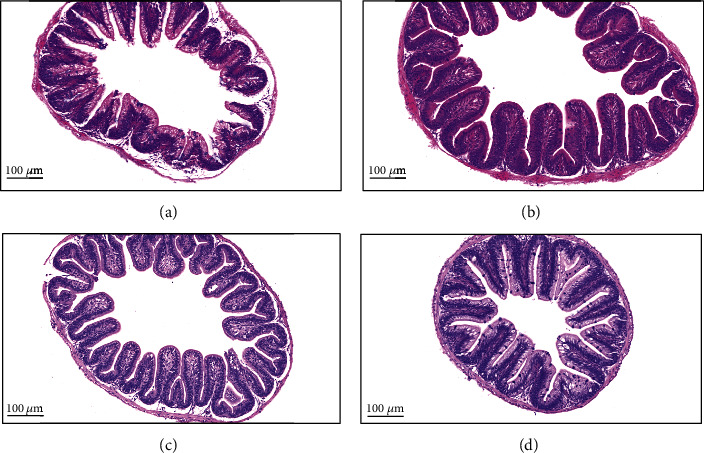
Effects of oat bran supplementation diet on the HE-stained sections of the gut of Nile tilapia: (a) control, (b) 5% OB, (c) 10% OB, and (d) 20% OB. OB: oat bran.

As shown in Figure 1, compared with those of the control group, the intestinal morphology of the experimental groups supplemented with oat bran was more complete, and the villi were neater and denser. The intestinal morphometric parameters are presented in Table 4. Supplementation with oat bran significantly increased the villus height and villus width compared to the control group (*P* < 0.05, Table 4). However, the villus height and villus width were significantly decreased in the 20% OB group compared to those in the 10% OB group (*P* < 0.05, Table 4). Additionally, the 5% OB and 10% OB groups had a thicker intestinal muscular layer than the control and 20% OB groups.

### 3.3. Intestinal Antioxidant Capacity

**Figure 2:**
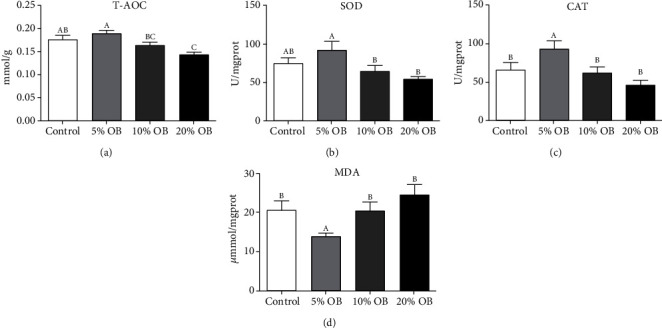
The intestinal antioxidant capacity of Nile tilapia fed with different diets. OB: oat bran. (a) T-AOC, (b) SOD, (c) CAT, and (d) MDA of Nile tilapia fed with different diets. Data are expressed as mean ± SEM of six replicates of each treatment. Values in each column with different superscripts have significant differences (*P* < 0.05).

As shown in Figure 2, T-AOC and SOD activities in the 5% OB group were higher than those in the control group, but the differences were not significant (*P* > 0.05). CAT activity was significantly higher and MDA content was significantly lower in the 5% OB group than those in the control group (*P* < 0.05). There was no significant difference between the 10% OB group and the control group (*P* < 0.05). In the three experimental groups, with the increase in oat bran content, the activities of T-AOC, SOD, and CAT showed a downward trend, while the MDA content showed an upwards trend.

### 3.4. Intestinal Immune Capacity

**Figure 3:**
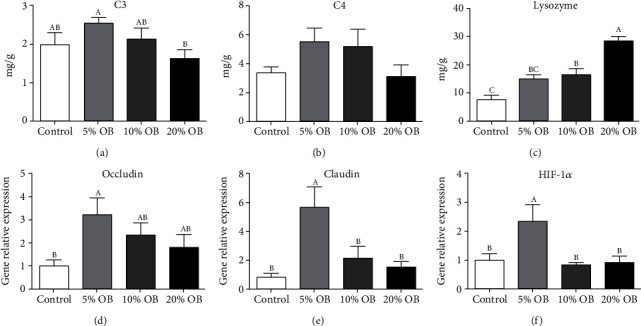
The intestinal immune capacity of Nile tilapia fed with different diets. OB: oat bran. (a) C3, (b) C4, (c) lysozyme, (d) occludin, (e) claudin, and (f) HIF-1*α* of Nile tilapia fed with different diets. Data are expressed as mean ± SEM of six replicates of each treatment. Values in each column with different superscripts have significant differences (*P* < 0.05).

**Figure 4:**
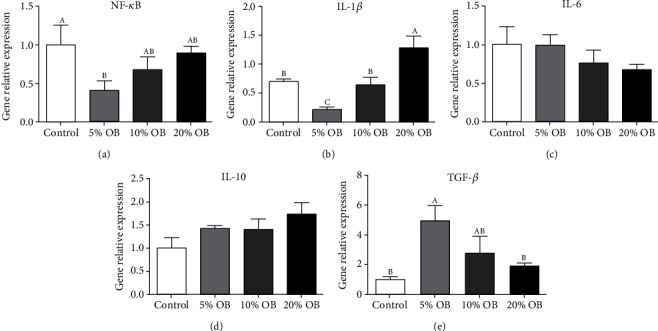
Intestinal expression levels of inflammatory/anti-inflammatory cytokine genes in Nile tilapia bass fed with different diets. OB: oat bran. (a) NF-*κ*B, (b) IL-1*β*, (c) IL-6, (d) IL-10, and (e) TGF-*β* of Nile tilapia fed with different diets. Data are expressed as mean ± SEM of six replicates of each treatment. Values in each column with different superscripts have significant differences (*P* < 0.05).

Figures 3 and 4 show the effects of oat bran supplementation on the intestinal immune capacity of Nile tilapia. As shown in Figure 3, the contents of C3 in the 5% OB group were significantly higher than the control group (*P* < 0.05). There was no significant difference in the contents of C4 among the four groups (*P* < 0.05). LYS activity in the 20% OB group was significantly higher than that in the other groups (*P* < 0.05). The relative gene expression levels of occludin in the 5% OB group were significantly higher than the control group (*P* < 0.05). The relative gene expression levels of claudin and Hif-1*α* in the 5% OB group were significantly higher than those in the other groups (*P* < 0.05).

As shown in Figure 4, the relative gene expression levels of inflammatory cytokine genes such as NF-*κ*B and IL-1*β* in the experimental group increased with increasing oat bran content. Compared with those in the control group, the relative expression levels of these two genes were significantly decreased in the 5% OB group, but they were not significantly different in the 10% OB group (*P* < 0.05). The relative expression of IL-6, also an inflammatory cytokine gene, decreased with increasing oat bran content. The relative expression level of anti-inflammatory cytokine genes in the experimental groups was higher than that in the control group, and the relative expression level of TGF-*β* in the 5% OB group was significantly higher than that in the control group (*P* < 0.05).

### 3.5. Composition and Diversity of Intestinal Microbiota

Table 5 shows the *α*-diversity index of Nile tilapia's intestinal microbiota. The Simpson index was higher in all groups supplemented with oat bran than in the control group, and the Simpson index of the 20% OB group was significantly higher than that in the control group (*P* < 0.05). The ACE index was highest in the 5% OB group and lowest in the 20% OB group (*P* < 0.05).

**Figure 5:**
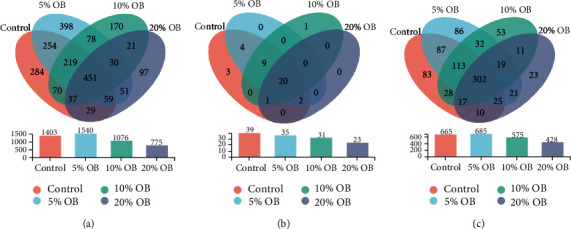
Comparison of intestinal microbiota diversity at the OUT (a), phylum (b), and genus (c) levels. OB: oat bran. In the Wayne diagram, the number of overlapping parts represents the number of common species in each group, while the number of nonoverlapping parts represents the number of species unique to the corresponding group.

The number of species of tilapia gut microorganisms at the OTU, phylum, and genus levels is shown in Figure 5. At the OTU level, the highest number of species was found in the 5% OB group. At the phylum level, there was a decreasing trend in the number of species as the amount of oat bran added to the diet increased. At the genus level, the four groups shared up to 302 species of microorganisms, while the 5% OB group had about 685 species in total and 86 species endemic to it, with more species in total and more species endemic to it than the other three groups.

**Figure 6:**
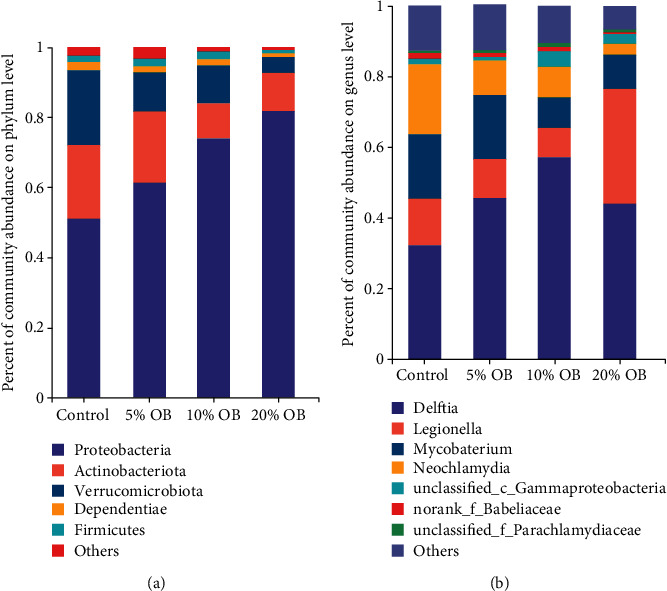
Intestinal bacterial composition in four groups at the phylum (a) and genus (b) levels. OB: oat bran. Only the top 6 most abundant (based on relative abundance) bacterial phyla and genus were shown. Other phyla and genus were all assigned as “others”.

**Figure 7:**
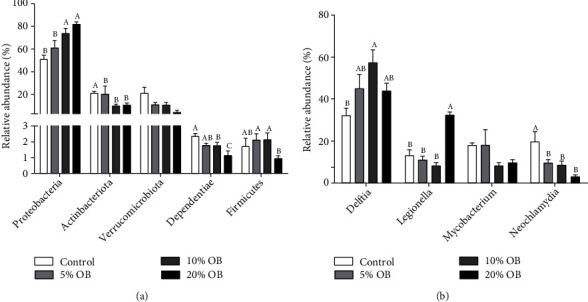
The relative abundance of the main phylum (a) and genus (b) in the intestinal microbiota of Nile tilapia fed with different diets. OB: oat bran. Data are expressed as mean ± SEM of six replicates of each treatment. Values in each column with different superscripts have significant differences (*P* < 0.05).

At the phylum level (Figures 6(a) and 7(a)), when oat bran was added, the species of intestinal flora decreased, and *Proteobacteria* was the most abundant phylum in Nile tilapia's intestinal microbiota, followed by *Actinobacteriota* and *Verrucomicrobiota*. The proportion of *Proteobacteria* increased with increasing oat bran addition, but *Verrucomicrobiota* showed the opposite trend, and the proportion of *Actinobacteriota* first decreased and then increased. The relative abundance of *Proteobacteria* was significantly higher in the 10% OB group and 20% OB group than in the control group and 5% OB group (*P* < 0.05). In contrast, the relative abundance of *Verrucomicrobiota* was significantly lower in the 20% OB group than in the other groups (*P* < 0.05). The abundance of *Actinobacteriota* was not significantly different among the groups.

At the genus level (Figures 6(b) and 7(b)), *Delftia*, *Legionella*, *Mycobacterium*, and *Neochlamydia* accounted for 80% of the intestinal microbiota. *Delftia* was the most abundant genus, with relative abundances of 32.19%, 45.17%, 57.52%, and 44.03% in the control, 5% OB, 10% OB, and 20% OB groups, respectively.

**Figure 8:**
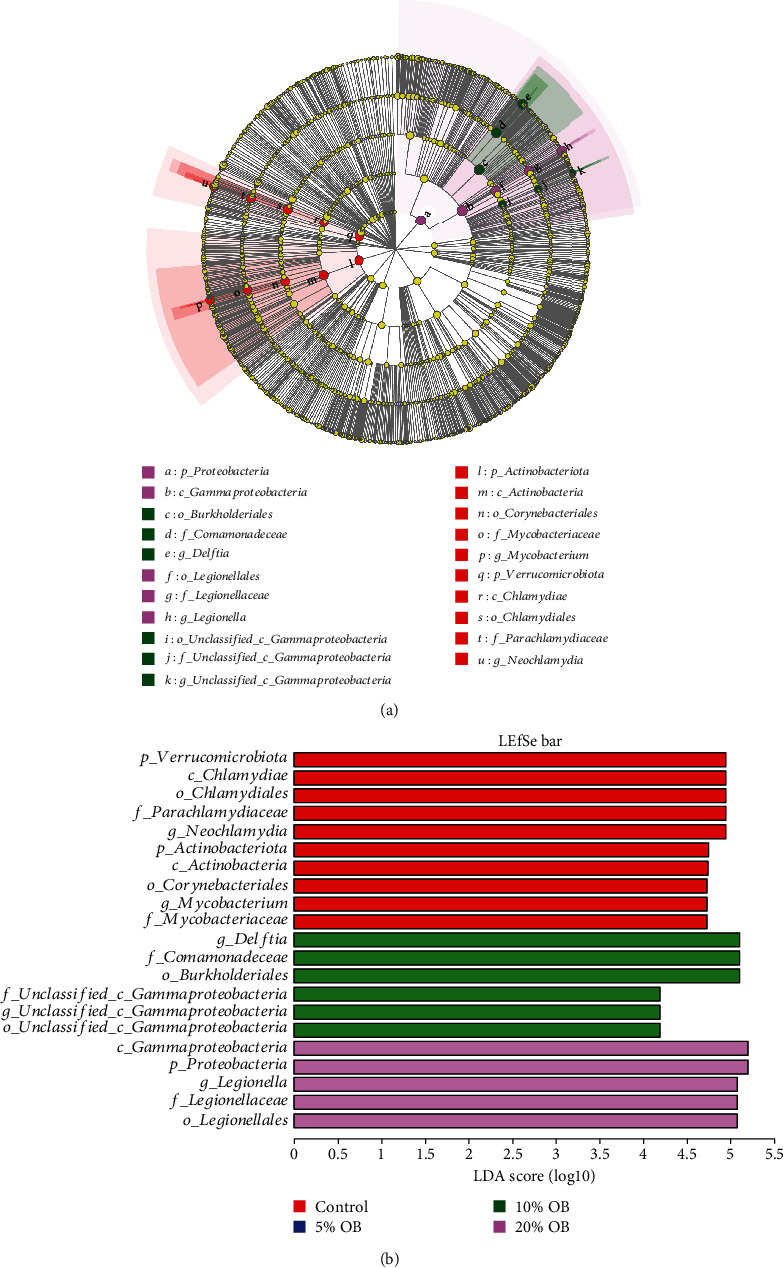
Linear discriminant analysis effect size of intestinal microorganisms in tilapia fed with different diets.

The effect of adding different levels of oat bran to the diet on the differences in the composition of the intestinal bacterial community of tilapia under copper ion stress is shown in Figure 8. At the phylum level, the differential bacteria in the control group were Actinobacteriota and Verrucomicrobiota; in the 20% OB group, the differential bacteria were Proteobacteria, while there were no differential bacteria in the 5% OB and 10% OB groups. At the genus level, the differential organisms in the control group were Mycobacterium and Neochlamydia; the differential organisms in the 10% OB group were Delftia and unclassified-c-Gammaproteobacteria; the differential organism in the 20% OB group was Legionella; and there were no differential organisms in the 5% OB group.

## 4. Discussion

The tolerance of fish to copper ions is affected by species and growth stage [16, 23]. Moreover, excessive copper ion concentrations can lead to abnormal expression of immune-related genes, change in antioxidant enzyme activity, and tissue damage in fish.

To mitigate the adverse effects of copper ions, oat bran was added to the diet for the experimental study. *β*-Glucan is the main component of soluble dietary fibre in oat bran, and the functions of oat bran are mainly realized by *β*-glucan. The effect of *β*-glucan on improving the growth performance and health of fish has been verified in pompano fish, juvenile Persian sturgeon, rainbow trout, and carp [24–27]. Therefore, we speculated that the effects of oat bran on the growth and intestinal health of tilapia may also be realized through *β*-glucan. In this study, *β*-glucan was not directly added to the diet but was added in the form of oat bran to comprehensively evaluate the growth effect of oat bran on tilapia.

The changes in FBR, WGR, SGR, and FCR reflected the effects of oat bran supplementation on the growth performance and feed efficiency of tilapia. Although the effect of *β*-glucan on fish growth is not completely clear, combined with existing studies, it can be inferred that a moderate amount of *β*-glucan has a positive effect on intestinal flora and the production of digestive enzymes [25]. *β*-Glucan is degraded by glucanases in the digestive glands to produce energy, allowing more protein to be used for growth and thus promoting growth [26]. After four weeks of feeding, the 20% OB group did not have the best effect, suggesting a dose effect of oat bran on the growth performance of Nile tilapia. *β*-Glucan is a type of nonstarch polysaccharides (NSPs). The high content of NSPs was made responsible for reduced digestibility of dry matter and energy in different species of fish. The effects of NSPs on nutrient digestibility could be dependent on the type of NSPs and their dietary concentration [28]. Therefore, adding excessive oat bran to the diet leads to an increase in *β*-glucan content, which has a negative impact on the growth performance of tilapia. On the other hand, we speculated that oat bran might contain other antinutritional factors that negatively affect tilapia growth. Due to oat bran's higher content of phytic acid (myoinositol hexaphosphate) as an antinutritional factor, consumption may cause adverse effects. Phytic acid is an antinutrient that reduces mineral and protein bioavailability [29]. It is also possible that the excess addition of oat bran increases digesta viscosity and limits interactions between intestinal nutrients and enzymes, thereby reducing nutrient digestion and absorption [10]. From the morphology of the tilapia intestine, we can see that the number and height of intestinal villus increased in both the 5% and 10% OB groups. Increasing the height of the villus means an increase in surface area for greater absorption of available nutrients [30, 31]. The muscular layer thickness in the 5% OB and 10% OB groups was significantly higher than that in the control group (*P* < 0.05). Enhanced muscular layer thickness has been reported to be associated with probiotic administration [32]. Therefore, dietary supplementation with an appropriate amount of oat bran could promote intestinal tract development and probiotic colonization in tilapia, which facilitates the absorption of nutrients by the intestine, thus promoting the growth of Nile tilapia.

Intestinal physical barrier integrity plays an important role in intestinal health [33]. In our study, the intestinal mucosa of the control group was slightly damaged, while the intestinal mucosa of the treatment group with oat bran was intact, indicating that the addition of oat bran had a positive effect on the protection of the intestinal mucosal layer. The increase in intestinal villus height not only improves nutrient absorption but is also directly related to the increase in epithelial cell turnover [33]. Epithelial cells also secrete cytokines and chemokines that regulate immune cells and resist the invasion of pathogens [34].

Fish immune health is a complex process. The immune response is closely related to the inflammatory response. An excessive immune response will lead to an inflammatory response. Immune cells and factors play an important role in the occurrence and extinction of inflammatory responses. Immune cells are activated to release inflammatory factors (such as TNF-*α*, IL-6, and IL-1*β*). On the other hand, the extinction of inflammation requires the release of anti-inflammatory factors by immune cells. In this study, the relative gene expression of NF-*κ*B and IL-1*β* was upregulated by increasing oat bran content, but when oat bran content did not exceed 10%, the levels of both proinflammatory factors were lower than those in the control group. The relative expression levels of two inflammatory cytokines (IL-10 and TGF-*β*) were higher in the oat bran groups than in the control group, and the changes in inflammatory factors could be explained by HIF-1*α*. HIF-1*α* is a major transcriptional regulator of the cellular response to hypoxia [35] and plays a critical role in immune and inflammatory responses [36]. This is because HIF-1*α* plays an important role in Treg-induced development, while IL-10 and TGF-*β* have been proven to be important factors affecting the development and function of Tregs [37]. It is speculated that HIF-1*α* upregulates the expression of IL-10 and TGF-*β* by promoting the development and proliferation of Tregs [38]. The proliferation of Tregs and the expression of anti-inflammatory factors have a protective effect on intestinal inflammation [37, 38]. There was a synergistic interaction between HIF and NF-*κ*B [39]. Moreover, HIF-1*α* can also activate the NF-*κ*B pathway, thus upregulating the expression of IL-1*β* and IL-6 and downregulating the expression of IL-10 and TGF-*β* [40, 41]. However, this result was not reflected in this study. It was speculated that the increase in HIF-1*α* in the 5% OB group was more involved in the Treg-induced development and production of IL-10 and TGF-*β*, which was reflected in the gene expression of occludin and claudin, and the gene expression of the two proteins in the 5% OB group was significantly higher than that in the other groups. Occludin and claudin are two proteins that form tight junctions in the intestine and play an important role in maintaining the integrity of the epithelial barrier function of the intestinal mucosa [42]. The injury of the intestinal epithelium of tilapia can be determined by monitoring its changes. Proinflammatory factors disrupt tight intestinal junctions, alter intestinal permeability, and affect intestinal barrier function. However, anti-inflammatory cytokines can increase tight junction protein expression and maintain the integrity of the intestinal barrier [43]. Combined with the changes in inflammatory factors, C3, C4, LYS, and the morphology of intestinal tissue sections, it can be inferred that adding oat bran can alleviate intestinal inflammation and improve intestinal immunity of tilapia, and 5% OB addition is the most effective.

Oxidative stress results from an imbalance between the production of reactive oxygen species and antioxidant defenses, in favor of the former. As one of the main products of lipid peroxidation, MDA can reflect the degree of lipid peroxidation and its antioxidant capacity. SOD and CAT are two important antioxidant enzymes that can effectively remove lipid peroxidation products from the body. T-AOC refers to the total antioxidant level composed of various antioxidant substances and antioxidant enzymes [44, 45]. Heavy metals in fish can disrupt the redox balance and alter antioxidant defenses [46]. In this study, T-AOC, SOD, CAT, and MDA were used to comprehensively evaluate the effects of oat bran supplementation on the intestinal antioxidant capacity of tilapia under copper ion stress. It turned out that the 5% OB had the highest antioxidant capacity, which was different from what we expected to see in the 10% OB group. This is probably because we added oat bran instead of *β*-glucan, so the dose effect of *β*-glucan did not show up, and oat bran also has many antinutritional factors. Tilapia had poor tolerance to diets with a high oat bran content; that is, with the increase in the oat bran ratio, the oxidative damage caused by copper ion stress in the 10% OB group was not alleviated but aggravated the oxidative damage in juvenile tilapia intestines. However, oxidative stress is the cause of many stress injuries and the pathophysiological basis of many diseases [47]. The 5% OB group reduced oxidative stress, which is probably one of the reasons why immune performance was improved [48].

When water is polluted by heavy metals, the organisms living in it will also be compromised. We can evaluate the intestinal health of Nile tilapia by the change in its intestinal microflora. After oat bran was added, the Simpson index was higher than that in the control group, indicating a decrease in community diversity. The species of the microbial community in the 5% OB group decreased at the phylum level but increased at the genus level. Combined with growth and antioxidant indices, it could be speculated that the decrease in community diversity might reduce the types of pathogenic bacteria and be more beneficial to intestinal health. Most of the gut microbiota of fish comprises *Actinobacteria*, *Bacteroidetes*, *Fusobacteria*, *Firmicutes*, and *Proteobacteria* cumulatively in phylum terms [49]. *Proteobacteria* played a dominant role in the intestinal flora in this study, followed by *Actinobacteria* and *Verrucomicrobia*. We found that the microbial community composition of the tilapia intestinal tract is very different from that reported in previous studies, which also indicates that the aquatic environment plays an important role in the factors affecting the intestinal microbial community [49, 50]. It has been reported that the reduction of *Firmicutes* is one of the main manifestations of intestinal inflammation [51]. Although *Firmicutes* were not the dominant bacteria in each group, the relative abundance of *Firmicutes* in the 5% OB and 10% OB groups was significantly higher than that in the control group and 20% OB group. This also suggests that adding oat bran can help reduce intestinal inflammation, but with a dose effect. We focused on *Delftia*, the genus with high relative abundance. *Delftia* is reported to degrade or transform a variety of organic and inorganic pollutants. *Delftia* reduces the toxic effects of heavy metals on organisms through bioaccumulation and transformation of heavy metals [52–54]. If we regard the intestinal tract of Nile tilapia as an environmental area polluted by heavy metals, the more *Delftia* bacteria there are, the stronger the ability to alleviate heavy metal pollution will be. Therefore, we innovatively considered *Delftia* as an indicator of heavy metal digestion in fish. The addition of oat bran to the diet could increase the relative abundance of *Delftia* in the microbiota and enhance the ability to alleviate copper ion stress.

## 5. Conclusion

In conclusion, dietary oat bran increased the weight gain and decreased the FCR of Nile tilapia, changed the intestinal microbial structure of Nile tilapia, and increased the relative abundance of *Delftia*, which can degrade heavy metals. Considering the effects on antioxidant capacity and immunity, only 5% of oat bran supplementation had positive effects. Taken together, our results indicated that 5% oat bran supplementation improved the growth performance and intestinal health of Nile tilapia exposed to copper ions.

## Figures and Tables

**Table 1:** Formulation and chemical composition of the experimental diets (dry matter, g/kg).

	Groups			
	Control	5% OB	10% OB	20% OB
*Ingredient composition*				
Flour	200.0	200.0	200.0	200.0
Rice bran meal	200.0	150.0	100.0	0.0
Oat bran^a^	0.0	50.0	100.0	200.0
Soybean meal	190.0	190.0	190.0	190.0
Rapeseed meal	110.0	114.0	116.0	122.0
Fish meal	80.0	80.0	80.0	80.0
Chicken power	120.0	120.0	120.0	120.0
Bentonite	4.5	3.0	3.6	2.0
Lys-HCl	2.0	1.6	1.2	0.4
Methionine	1.0	0.8	0.5	0.0
Choline chloride (50%)	2.0	2.0	2.0	2.0
Ca(H_2_PO_4_)_2_	20.0	20.0	20.0	20.0
Soybean oil	60.0	58.1	56.2	52.5
VC phosphate	0.5	0.5	0.5	0.5
Fish premix (1%)^b^	10.0	10.0	10.0	10.0
Total	1000	1000	1000	1000

*Chemical composition*				
Moisture (%)	4.74	5.90	6.57	5.53
Crude protein (%)	34.45	34.23	33.88	34.41
Crude fat (%)	10.07	10.21	9.71	9.83
Crude ash (%)	10.08	9.29	8.78	7.99

OB: oat bran. ^a^Provided by Zhenfang Enterprise Management (Shanghai) Co., Ltd. It contains 15% crude protein, 7.5% crude fat, and 10% *β*-glucan. ^b^Provided by Beijing Sino-Norway Joint Aquaculture Technology Co., Ltd. The product meets NRC standards. Other ingredients are produced by the Chinese Academy of Agricultural Sciences.

**Table 2:** Primer sequences for qPCR.

Gene	Forward primer (5′-3′)	Reverse primer (5′-3′)
*β*-Actin	CAGCAAGCAGGAGTACGATGAGTC	GTATGAGAAATGTGTGGTGTGTGGTTG
Occludin	GGAGGAAAGCCGCAGTGTTCAG	GTCGTAGGCATCGTCATTGTAGGAG
Claudin	GTCTGTTTCTGGGCGTGGTGTC	ACTCCGACTGACTCCTCATCTTCC
HIF-1*α*	AAGCAGACCGCAGATGTGAAGC	TCCTCCTTCTCCAGTTCAGCCTTC
NF-*κ*B	GCAGGATTACGAGCCTTGGAGAAC	ATTGAGGAACGGGTGATTGTGAGAC
IL-1*β*	ACAAGGATGACGACAAGCCAACC	GGACAGACATGAGAGTGCTGATGC
IL-6	ATAGCAAGCATCTACACGCATCTCC	GGGCTGCCAGGGAATTGTAAGTC
IL-10	GCTTCCCCGTCAGGCTCAA	CTGTCGGCAGAACCGTGTC
TGF-*β*	GCCCATCAGCTCACCTACAAATCC	ATGACCGAAGAGGAGGAAGAGGAAG

**Table 3:** Effects of oat bran supplementation on growth performance and feed utilization of Nile tilapia.

Parameters	Experimental diets			
	Control	5% OB	10% OB	20% OB
IBW (g)	1.10 ± 0.00	1.10 ± 0.00	1.10 ± 0.00	1.10 ± 0.00
FBW (g)	2.46 ± 0.07^b^	2.58 ± 0.02^ab^	2.62 ± 0.06^a^	2.51 ± 0.13^ab^
WGR (%)	123.06 ± 5.17^b^	135.33 ± 2.15^ab^	138.67 ± 5.05^a^	128.38 ± 11.86^b^
FCR	1.50 ± 0.05	1.36 ± 0.05	1.37 ± 0.09	1.43 ± 0.13
SGR (%)	2.86 ± 0.08^b^	3.06 ± 0.03^ab^	3.11 ± 0.08^a^	2.95 ± 0.19^b^
SR (%)	93.33 ± 5.77	95 ± 5.00	96.67 ± 2.89	83.33 ± 20.82

OB: oat bran; IBW: initial body weight; FBW: final body weight; WGR: weight gain ratio; FCR: feed coefficient ratio; SGR: specific growth rate; SR: survival rate. Data are expressed as mean ± SEM of six replicates of each treatment. Different superscripts in the same row mean significant differences (*P* < 0.05).

**Table 4:** The posterior intestinal morphology of Nile tilapia fed with different diets.

Items	Control	5% OB	10% OB	20% OB
Villus height (*μ*m)	163.93 ± 2.96^c^	202.6 ± 3.87^b^	214.8 ± 3.29^a^	194.73 ± 1.37^b^
Villus width (*μ*m)	71.17 ± 1.21^c^	81.93 ± 0.77^a^	84.17 ± 0.73^a^	76.3 ± 1.59^b^
Muscular layer thickness (*μ*m)	21.57 ± 0.43^b^	24.17 ± 0.66^a^	24.27 ± 0.65^a^	22.7 ± 0.19^ab^

OB: oat bran. Data are expressed as mean ± SEM of six replicates of each treatment. Different superscripts in the same row mean significant differences (*P* < 0.05).

**Table 5:** Simpson and ACE indices of the intestine microbiota of Nile tilapia fed with different diets.

Parameters	Control	5% OB	10% OB	20% OB
Simpson	0.19 ± 0.02^b^	0.3 ± 0.02^ab^	0.37 ± 0.05^ab^	0.3 ± 0.02^a^
ACE	572.94 ± 76.92^ab^	665.46 ± 61.16^a^	437.51 ± 42.03^bc^	356.36 ± 18.56^c^

OB: oat bran. Data are expressed as mean ± SEM of six replicates of each treatment. Different superscripts in the same row mean significant differences (*P* < 0.05).

## Data Availability

The data presented in this study are available in this article.
